# Pocket ACEs: Discovering new function within an old player

**DOI:** 10.3389/fphys.2023.1151908

**Published:** 2023-03-10

**Authors:** Matthew Leong, Xiaomo Li, Manita Chaum

**Affiliations:** Cedars Sinai Medical Center, Los Angeles, CA, United States

**Keywords:** ACE, macrophage, hemato- and immunological effects, neutrophil, anti inflammation, immunmodulation, RAS

## Abstract

Angiotensin-converting enzyme (ACE) is canonically known for its role in the renin-angiotensin system (RAS) where its conversion of angiotensin I (Ang I) to the bioactive peptide angiotensin II (Ang II) helps to regulate blood pressure, electrolyte, and volume homeostasis. Further studies on ACE have shown that its enzymatic activity is relatively non-specific and functions outside of the RAS axis. Of the multiple systems it has been implicated in, ACE has been found to play an important role in the development and modulation of hematopoiesis and the immune system, both through the RAS and independently of the RAS axis.

## Introduction

Angiotensin-converting enzyme (ACE) is a type-I cell surface zinc metallopeptidase with two functional catalytic domains towards the N- and C-terminal and is a crucial component of the renin-angiotensin system (RAS). ACE is responsible for conversion of angiotensin I (Ang I) to the bioactive peptide angiotensin II (Ang II) (Peart, 1975; Nishimura, 2017). Angiotensin II binds to angiotensin II type 1 (AT1) and type 2 (AT2) receptors, as well as G protein-coupled receptors, which increases sodium reabsorption in the kidney, stimulates release of aldosterone in the adrenal cortex, vasoconstriction in systemic arterioles, and triggers thirst, release of antidiuretic hormone, and suppresses baroreceptor response in the brain (Fountain and Lappin, 2022). Not only is ACE critical for maintaining blood pressure through these complex interactions, the RAS has many additional functions including roles in apoptosis and fibrosis (Laghlam et al., 2021). Further studies on ACE have shown that its enzymatic activity is relatively non-specific and additionally functions outside of the RAS axis. Through both its role in the RAS and independent of the RAS pathway, ACE has been found to play an important role in the development and modulation of hematopoiesis and the immune system.

## ACE’s role in myelopoiesis

ACE has been shown to be involved in the development of hematopoiesis in humans. ACE expression in embryonic body cells is linked with hematopoietic potential, even more so than other markers such as CD34 (Jokubaitis, 2008). ACE is continually expressed in hematopoietic stem cells from all human embryonic, fetal, and adult hematopoietic tissues.

Part of ACE’s regulation over myelopoiesis is invoked through the RAS. Action of Ang II through the AT1 receptors is important for terminal myeloid differentiation and proliferation of CD34^+^ hematopoietic stem cells. ACE knock-out mice shows decreased segmented neutrophils but increase in progenitor cell types, suggesting issues with terminal granulopoiesis (Lin, 2011). Blockage of AT1 receptors interferes with dendritic cell maturation while addition of Ang II stimulates dendritic cell maturation (Nahmod, 2003). Consequently, ACE inhibition has been shown to cause myelosuppression (Chisi et al., 1999).

Angiotensin-(1-7) (Ang 1-7), another component of the RAS system produced through breakdown of Ang I or Ang II, has also been shown to cause pan-lineage proliferation which accelerated hematopoietic recovery in mice after irradiation (Ellefson, 2004; Heringer-Walther, 2009). Ang 1-7 role in myelopoiesis has led to pharmaceutical formulations being tested for accelerated engraftment in post-stem cell transplantation in humans.

ACE can additionally exert influence over myelopoiesis through its non-RAS related enzymatic function. ACE is capable of degrading substance P and thereby can regulate its level within the bone marrow (Skidgel and Erdös, 2004). While typically associated with its role in pain sensation, substance P is present in bone marrow both through transport from terminals of projected neurofibrils and production by native bone marrow cells such as macrophages and eosinophils (Johnson and Torres, 1988; Weinstock and Blum, 1989; Pascual and Bost, 1990; Bost et al., 1992; Rameshwar et al., 1997). Substance P’s primary endogenous receptor, neurokinin 1 (NK-1), is expressed on lymphocytes, macrophages, CD34 cells, and endothelial cells (Payan et al., 1984; Scicchitano et al., 1987; Greeno et al., 1993; Rameshwar et al., 1996). Through these cells, substance P has been shown to stimulate many growth factors, including IL-1, IL-3, and GM-SCF, with recent evidence showing that substance P may be able to function independently as a myeloid growth factor (Rameshwar et al., 1993; Rameshwar et al., 1995).

Acetyl-Ser-Asp-Lys-Pro (Ac-SDKP) is another substrate degraded by ACE. Studies looking at both ACE knock-out and ACE inhibitors have shown that decreased ACE activity significantly increased the serum and bone marrow levels of Ac-SDKP (Azizi, 1996; Li, 1997). Ac-SDKP has an inhibitory effect on hematopoietic progenitor proliferation; ACE, through its hydrolysis of Ac-SDKP, has been shown to recruit stem cells into S-phase (Lenfant, 1989; Bonnet, 1993; Rousseau-Plasse et al., 1996).

Many studies looking at the global effect of ACE inhibition on hematopoiesis have occurred in the setting of radiation, in which ACE inhibition leads to bi-phasic modulation of ACE inhibition on hematopoiesis. Short-term ACE inhibition, on the scale of days, impairs the G_0_ to G_1_ transition, delaying hematopoiesis which has radioprotective effects and is at least partially modulated through the inhibition of the RAS and increased AcSDKP concentration. Longer inhibition, on the scale of weeks after radiation exposure and initiation of ACE inhibitors, leads to increased progenitor proliferation compared to untreated controls (Charrier, 2004; Davis, 2010). Outside of radiation, ACE inhibition and hematopoiesis have been studied in the setting of myocardial infarction. This study showed that ACE inhibition led to retention of myeloid precursors in the bone marrow and reduction in circulating inflammatory cells, particularly monocytes/macrophages, even weeks after treatment (Rudi, 2021). The variable effect of ACE inhibition on hematopoiesis depending on time frame and injury etiology emphasizes the complex and multifactorial role of ACE in myelopoiesis.

## ACE’s modification of immune cell function and maturation

**FIGURE 1:**
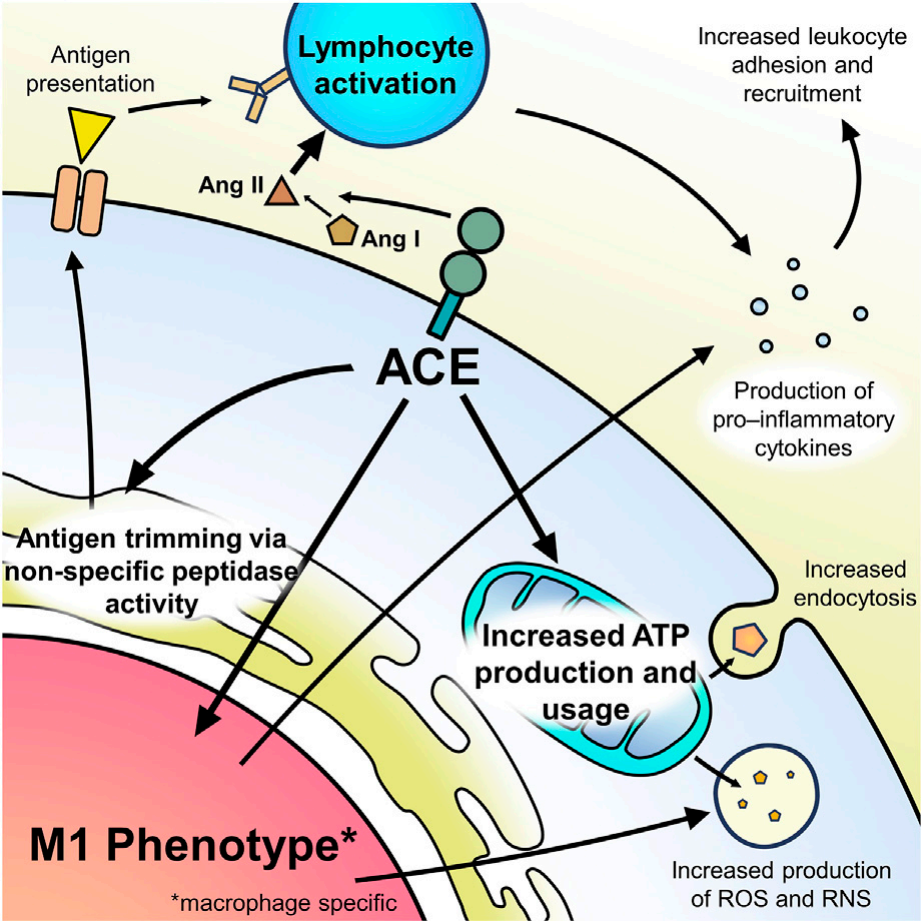
Summary of ACE’s effect on immune cell function.

ACE has many interactions with the different immune cell types and, through them, is an important modulator of the immune response (Figure 1). In general, ACE is a pro-inflammatory modulator which regulates chemokines and adhesion molecules (Ruiz-Ortega, 1998). This explains why ACE levels are increased in some conditions with chronic inflammation, such as sarcoidosis and other granulomatous diseases. ACE’s importance in modulating the immune response can be seen in recent studies showing that lower serum ACE levels are associated with impaired host antiviral response to COVID-19 as well as studies showing increased risk of infection with ACE inhibitor use (Pouwels et al., 2013; Dial et al., 2014; Pouwels et al., 2014; Chen, 2021).

ACE is near ubiquitously expressed in tissues throughout the body including mononuclear cells in the peripheral blood, although expression is relatively low compared to other sites such as the small intestine and testes (Harmer et al., 2002). There is trace ACE expression in monocytes which drastically increases to high expression as they differentiate into macrophages (Costerousse et al., 1993). T-cells have intermediary expression of ACE with increased expression during processes associated with inflammation (Costerousse et al., 1993; Ulrich, 2006; Coppo, 2022). While previous studies suggested that ACE was not expressed in B-cells, recent studies using flow cytometry have shown expression of ACE in nearly all B-lymphocytes, although the relative level of expression is not known. While T-cells also showed near complete expression of ACE, in non-lymphoid cells only around 57% of cells expressed ACE (Bueno et al., 2023).

Neutrophils are hallmark cells of acute inflammation and a key component of the innate immune response. Numerous studies show that ACE plays a crucial and multifunctional role in neutrophil response. Inhibition of ACE leads to a reduction in neutrophil recruitment to sites of injury. Studies showed that, in response to methicillin-resistant *Staphylococcus aureus* (MRSA) challenge, ACE knocked-out mice had lower bacterial resistance as indicated by larger skin lesions and higher bacterial burden. Conversely, a line of mice which had overexpression of ACE in neutrophils (NeuAce mice) showed enhanced bacterial resistance. An explanation for these differences is that ACE expression is directly correlated to neutrophil production of reactive oxygen species (ROS) (Khan, 2017). Interestingly, these differences were nullified through use of ACE inhibitors but persisted through use of losartan, suggesting that these effects are mediated independently of the RAS axis.

Macrophage function is influenced significantly by ACE. ACE is upregulated during monocyte differentiation into macrophages and seems to have an important role in macrophage functional maturation (Kohlstedt et al., 2011). Studies in ACE10/10 mice, which have increased ACE expression in macrophages, showed that these mice had increased bacterial resistance, as seen by smaller lesions and lower wound bacterial counts after exposure to MRSA. Macrophages from these mice showed increased nitric oxide (NO) production (Okwan-Duodu, 2010). The role of the RAS in this process is not well-defined—studies have shown that, like neutrophils, the difference in bacterial resistance is abrogated with ACE inhibitor use but persist with losartan use, suggesting a mechanism independent of the RAS; however, other studies show that losartan use led to functional immaturity of macrophages leading to bacterial susceptibility which was rescued with Ang II supplementation (Lin, 2011). Interestingly enough, the ACE10/10 mice also showed increased tumor resistance which seemed to be mediated by tumor epitope-specific CD8^+^ T-cells. Studies looking at mice with independently knocked out N- and C-domains of ACE in myeloid cells revealed that this tumor resistance could be attributed to the ACE C-domain, which seemed to induce macrophages to assume an M1 phenotype (Khan, 2019). ACE 10/10 macrophages significantly mitigated cognitive defects in Alzheimer’s disease mouse models through proposed increased ability to cleave and clear Aβ peptides (Bernstein, 2014).

ACE also has important functions in mediating endocytosis and T-cell stimulation properties of dendritic cells; however, empiric observations on ACE’s role in both dendritic cells and macrophages is likely linked to its shared role on preparing antigens for presentation on major histocompatibility complex (MHC) class I peptides. Studies have shown that knocking out or inhibiting ACE in mice significantly altered the repertoire of MHC class I peptides, suggesting that the non-specific peptidase activity of ACE functions in trimming peptides for display and might explain why there is impaired T-cell stimulation with decreased ACE activity (Shen et al., 2008; Shen, 2011).

An important mediator of ACE is through its influence over cellular ATP. Recent studies have shown that ACE C-domain catalytic expression is associated with upregulation of numerous proteins, including electron transport chain proteins NDUFB8, ATP5A, and ATP5β and has been associated with an increase in ATP production. This increase in ATP has subsequently been linked to increase in phagocytosis and superoxide production, providing a mechanism for the functional maturation of these myeloid cells. ATP-upregulation in this context is counteracted by use of ACE-inhibitor but not angiotensin II AT1 receptor antagonists, likewise suggesting that this change is mediated outside of the RAS axis (Cao, 2020).

Despite its near universal expression in lymphocytes, ACE’s effect in lymphocytes has not been extensively studied (Bueno et al., 2023). Beyond its role as a peptidase in activation of T-cells through antigen presenting cells (APCs), Ang II production through the RAS have been shown to activate T-lymphocytes, increase expression of tissue homing markers, and induce lymphocytic production of TNF-alpha (Hoch, 2009).

## Conclusion

ACE is an exceptionally important enzyme for its role in mediating homeostasis and blood pressure; however, as more studies are performed, ACE has become deeply implicated in the immune response due to regulation of myelopoiesis and immune cell functional maturation. ACE accomplishes this both through its role in the RAS as well as its independent peptidase functions. Although there are complex and multifactorial interactions with ACE and the immune system, understanding of the mechanisms and extent of ACE’s influence has yet to be fully explored.
